# Supplementary material for: OGG1 and MUTYH repair activities promote telomeric 8-oxoguanine induced senescence in human fibroblasts
Source: Nat Commun. 2025 Jan 21;16:893. doi: 10.1038/s41467-024-55638-4 (PMC11751180; doi:10.1038/s41467-024-55638-4)
Supplement: Supplementary file 1 — Supplementary Information [file 41467_2024_55638_MOESM1_ESM.pdf]

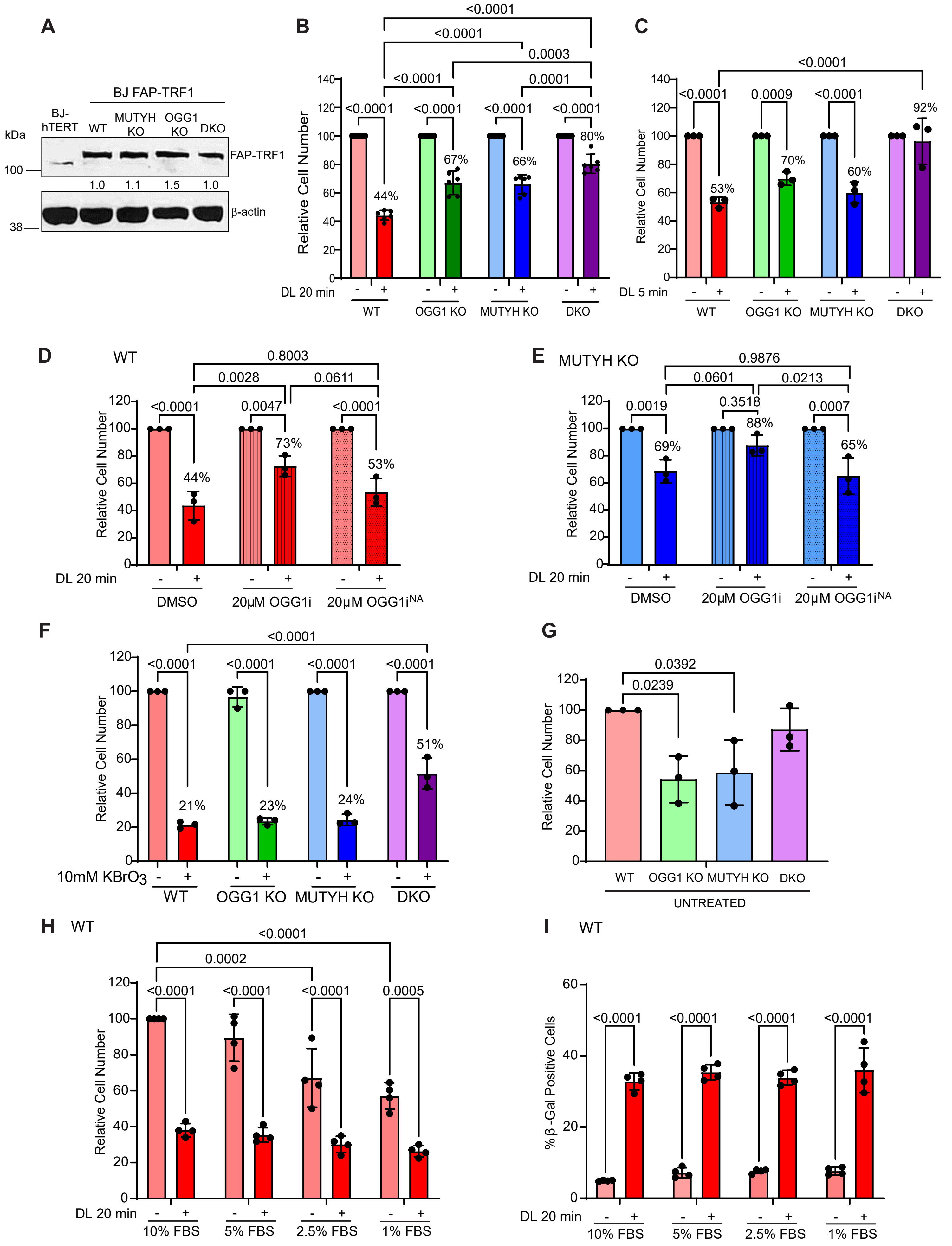

A
B
C
D
E
F
G
H
I
BJ FAP-TRF1
BJ-hTERT
WT
MUTYH KO
OGG1 KO
DKO
kDa
100
38
FAP-TRF1
β-actin
1.0 1.1 1.5 1.0
Relative Cell Number
DL 20 min
DL 5 min
WT
OGG1 KO
MUTYH KO
DKO
<0.0001
0.0003
0.0001
0.0009
0.8003
0.0028
0.0611
0.0047
MUTYH KO
0.9876
0.0601
0.0213
0.0019
0.3518
0.0007
DMSO
20μM OGG1i
20μM OGG1i^{NA}
10mM $KBrO_3$
UNTREATED
0.0392
0.0239
0.0002
0.0005
10% FBS
5% FBS
2.5% FBS
1% FBS
% β-Gal Positive Cells

SUPPLEMENTARY INFORMATION

Figure legends

Supplementary Figure 1. OGG1 and MUTYH deficiency reduces sensitivity to acute oxidative telomere damage. Related to Fig. 1.

A TRF1 immunoblot showing FAP-mCer-TRF1 expression in BJ FAP-TRF1 cells; indicated as FAP-TRF1. β-actin used as a loading control.

B Statistical comparison of cell counts for all BJ FAP-TRF1 cell lines obtained 4 days after recovery from 20 min dye + light (DL), relative to untreated cells from **Fig 1B**. Data are mean ± SD from six independent experiments; *P*-values were obtained using two-way ANOVA.

C Cell counts of BJ FAP-TRF1 cell lines obtained 4 days after recovery from 5 min DL, relative to untreated cells. Data are mean ± SD from three independent experiments; *P*-values were obtained using two-way ANOVA.

D-E Cell counts of WT (**D**) and MUTYH KO (**E**) obtained 4 days after recovery from 20 min DL in the presence of DMSO or 20 μM of OGG1 inhibitor TH5487 (OGG1i) or 20 μM of inactive OGG1 inhibitor ($OGG1i^{NA}$), relative to cells not treated with DL. Data are mean ± SD from three independent experiments; *P*-values were obtained using two-way ANOVA.

F Cell counts of BJ FAP-TRF1 cell lines obtained 4 days after recovery from 1h of 10mM $KBrO_3$ treatment, relative to untreated cells. Data are mean ± SD from three independent experiments; *P*-values were obtained using two-way ANOVA.

G Cell counts of untreated cell lines obtained after 4 days of growth, normalized to WT cells. Data are mean ± SD from three independent experiments; *P*-values were obtained using ordinary one-way ANOVA with Tukey's multiple comparisons test.

H Cell counts of WT cells cultured in the indicated FBS concentrations, obtained 4 days after recovery from 20 min DL, relative to WT untreated cells grown in standard 10% FBS. Data are mean ± SD from four independent experiments; *P*-values were obtained using two-way ANOVA.

I Percent β-galactosidase positive WT cells cultured in the indicated FBS concentrations. Data are mean ± SD from four independent experiments; *P*-values were obtained using two-way ANOVA.

Only comparisons yielding significant p-values are shown, except in panels **D-E**.

Source data are provided as a Source Data file.

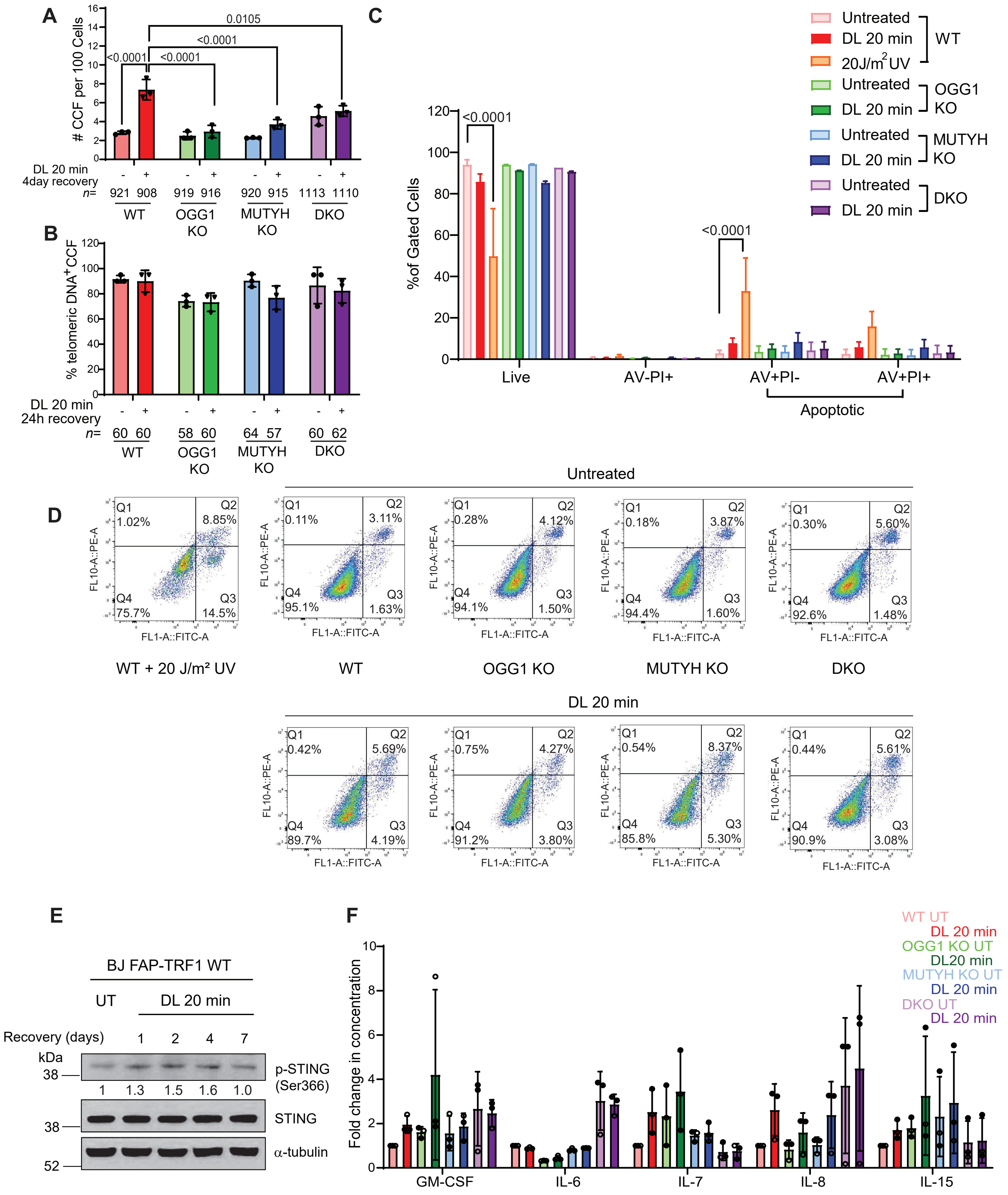
A
CCF per 100 Cells
<0.0001
<0.0001
<0.0001
0.0105
DL 20 min
4day recovery
n=
921 908
919 916
920 915
1113 1110
WT
OGG1 KO
MUTYH KO
DKO
B
% telomeric DNA+CCF
DL 20 min
24h recovery
n=
60 60
58 60
64 57
60 62
WT
OGG1 KO
MUTYH KO
DKO
C
Untreated
DL 20 min
20J/m2 UV
WT
Untreated
DL 20 min
OGG1 KO
Untreated
DL 20 min
MUTYH KO
Untreated
DL 20 min
DKO
%of Gated Cells
<0.0001
<0.0001
Live
AV-PI+
AV+PI-
AV+PI+
Apoptotic
D
Untreated
Q1 1.02% Q2 8.85% Q4 75.7% Q3 14.5%
Q1 0.11% Q2 3.11% Q4 95.1% Q3 1.63%
Q1 0.28% Q2 4.12% Q4 94.1% Q3 1.50%
Q1 0.18% Q2 3.87% Q4 94.4% Q3 1.60%
Q1 0.30% Q2 5.60% Q4 92.6% Q3 1.48%
FL10-A::PE-A
FL1-A::FITC-A
WT + 20 J/m² UV
WT
OGG1 KO
MUTYH KO
DKO
DL 20 min
Q1 0.42% Q2 5.69% Q4 89.7% Q3 4.19%
Q1 0.75% Q2 4.27% Q4 91.2% Q3 3.80%
Q1 0.54% Q2 8.37% Q4 85.8% Q3 5.30%
Q1 0.44% Q2 5.61% Q4 90.9% Q3 3.08%
E
BJ FAP-TRF1 WT
UT
DL 20 min
Recovery (days) 1 2 4 7
kDa
38
p-STING (Ser366)
1 1.3 1.5 1.6 1.0
38
STING
52
α-tubulin
F
Fold change in concentration
WT UT
DL 20 min
OGG1 KO UT
DL20 min
MUTYH KO UT
DL 20 min
DKO UT
DL 20 min
GM-CSF
IL-6
IL-7
IL-8
IL-15

Supplementary Figure 2. Glycosylase activity enhances telomeric 8oxoG-induced cytoplasmic DNA. Related to Fig. 2.

A Quantification of cytoplasmic DNA species (CCFs) 4 days after recovery from 20 DL. Data are mean ± SD from three independent experiments; *n* = total number of nuclei scored. *P*-values were obtained using two-way ANOVA.

B Quantification of CCFs positive for telomeric DNA staining 24 h after recovery from 20 min DL. Data are mean ± SD from three independent experiments; *n* = total number of CCFs analyzed.

C Percent of cells positive for annexin V (AV), propidium iodide (PI), or both, 4 days after indicated treatments. Data are the mean ± SD from two independent experiments; *P*-values were obtained using two-way ANOVA.

D Representative scatterplots of Annexin V (x-axis) and propidium iodide (y-axis) staining of cells 4 days after the indicated treatments.

E Immunoblot of phosphorylated STING and total STING in BJ FAP-TRF1 WT cells following the indicated recovery days from 20 min DL. α-tubulin used as a loading control.

F SASP analysis of BJ FAP-TRF1 cells 7 days post-treatment with DL 20 min. Concentration normalized to the final cell number in each sample. Data are presented as fold changes. Data are mean ± SD from two technical replicates of one experiment (dark circles) and one replicate from an independent experiment (open circles). Source data are provided as a Source Data file.

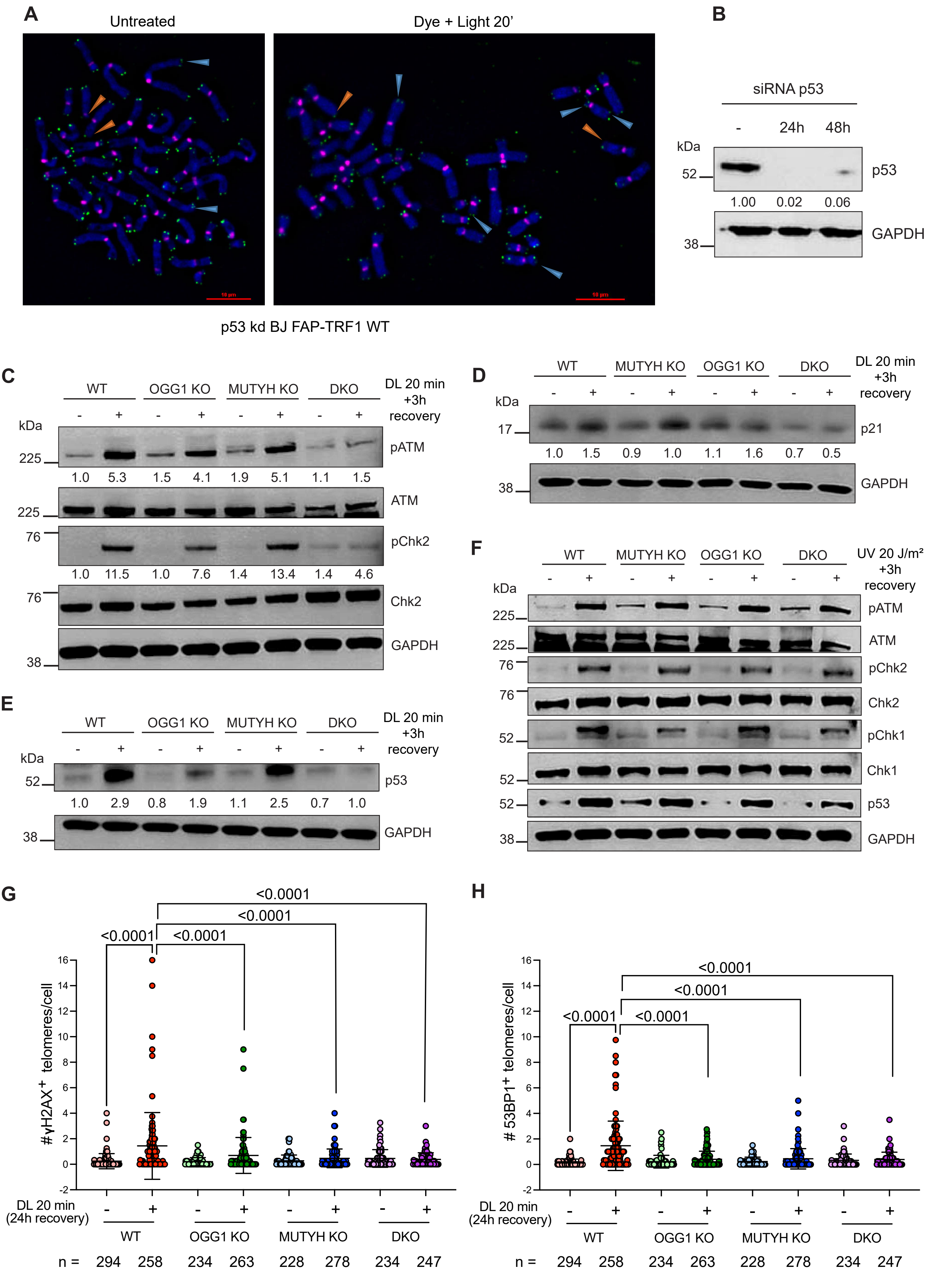

A
Untreated
Dye + Light 20'
p53 kd BJ FAP-TRF1 WT
B
siRNA p53
-
24h
48h
kDa
52
p53
1.00
0.02
0.06
38
GAPDH
C
WT
OGG1 KO
MUTYH KO
DKO
DL 20 min +3h recovery
kDa
225
pATM
1.0 5.3 1.5 4.1 1.9 5.1 1.1 1.5
225
ATM
76
pChk2
1.0 11.5 1.0 7.6 1.4 13.4 1.4 4.6
76
Chk2
38
GAPDH
D
WT
MUTYH KO
OGG1 KO
DKO
DL 20 min +3h recovery
kDa
17
p21
1.0 1.5 0.9 1.0 1.1 1.6 0.7 0.5
38
GAPDH
E
WT
OGG1 KO
MUTYH KO
DKO
DL 20 min +3h recovery
kDa
52
p53
1.0 2.9 0.8 1.9 1.1 2.5 0.7 1.0
38
GAPDH
F
WT
MUTYH KO
OGG1 KO
DKO
UV 20 J/m² +3h recovery
kDa
225
pATM
225
ATM
76
pChk2
76
Chk2
52
pChk1
52
Chk1
52
p53
38
GAPDH
G
<0.0001
<0.0001
<0.0001
<0.0001
#γH2AX+ telomeres/cell
DL 20 min (24h recovery)
WT
OGG1 KO
MUTYH KO
DKO
n = 294 258 234 263 228 278 234 247
H
<0.0001
<0.0001
<0.0001
<0.0001
53BP1+ telomeres/cell
DL 20 min (24h recovery)
WT
OGG1 KO
MUTYH KO
DKO
n = 294 258 234 263 228 278 234 247

Supplementary Figure 3. Glycosylase deficiency suppresses telomeric 8oxoG-induced replication stress. Related to Fig. 3.

A Representative images of telomere FISH on metaphase spreads in p53 kd BJ FAP-TRF1 WT cells untreated or treated with 20 min DL**.** Green foci are telomeres and pink foci are CENPB centromeres. Scale bars = 10 µm. Orange and blue arrowheads point to signal free ends and fragile telomeres, respectively.

B Immunoblot of p53 in WT cells transiently transfected with siRNAs against p53 and cultured for the indicated times. Numbers below p53 blot represent normalized protein expression. GAPDH used as a loading control.

C Immunoblot of indicated proteins in cells untreated or treated with 20 min DL and recovered 3h. Numbers below pATM and pChk2 blots represent normalized protein expression as the average of three independent experiments. GAPDH used as a loading control.

D Immunoblot of indicated proteins in cells untreated or treated with 20 min DL and recovered 3h. Numbers below p21 blot represent normalized protein expression as the average of three independent experiments. GAPDH used as a loading control.

E Immunoblot of indicated proteins in cells untreated or treated with 20 min DL and recovered 3h. Numbers below p53 blot represent normalized protein expression as the average of three independent experiments. GAPDH used as a loading control.

F Immunoblot of indicated proteins in cells untreated or treated with 20 J/m^2 UVC light, and recovered 3h; pATM, pChk2 and pChk1 indicate phosphorylated forms; GAPDH used as a loading control.

G-H Quantification of the number of γH2AX positive (**G**) or 53BP1 positive (**H**) telomeres per cell, 24 h after no treatment or 20 min DL. Data represent the mean ± SD from four independent experiments, from *n*= total number of nuclei analyzed per experiment; *P*-values were obtained using two-way ANOVA.

Source data are provided as a Source Data file.

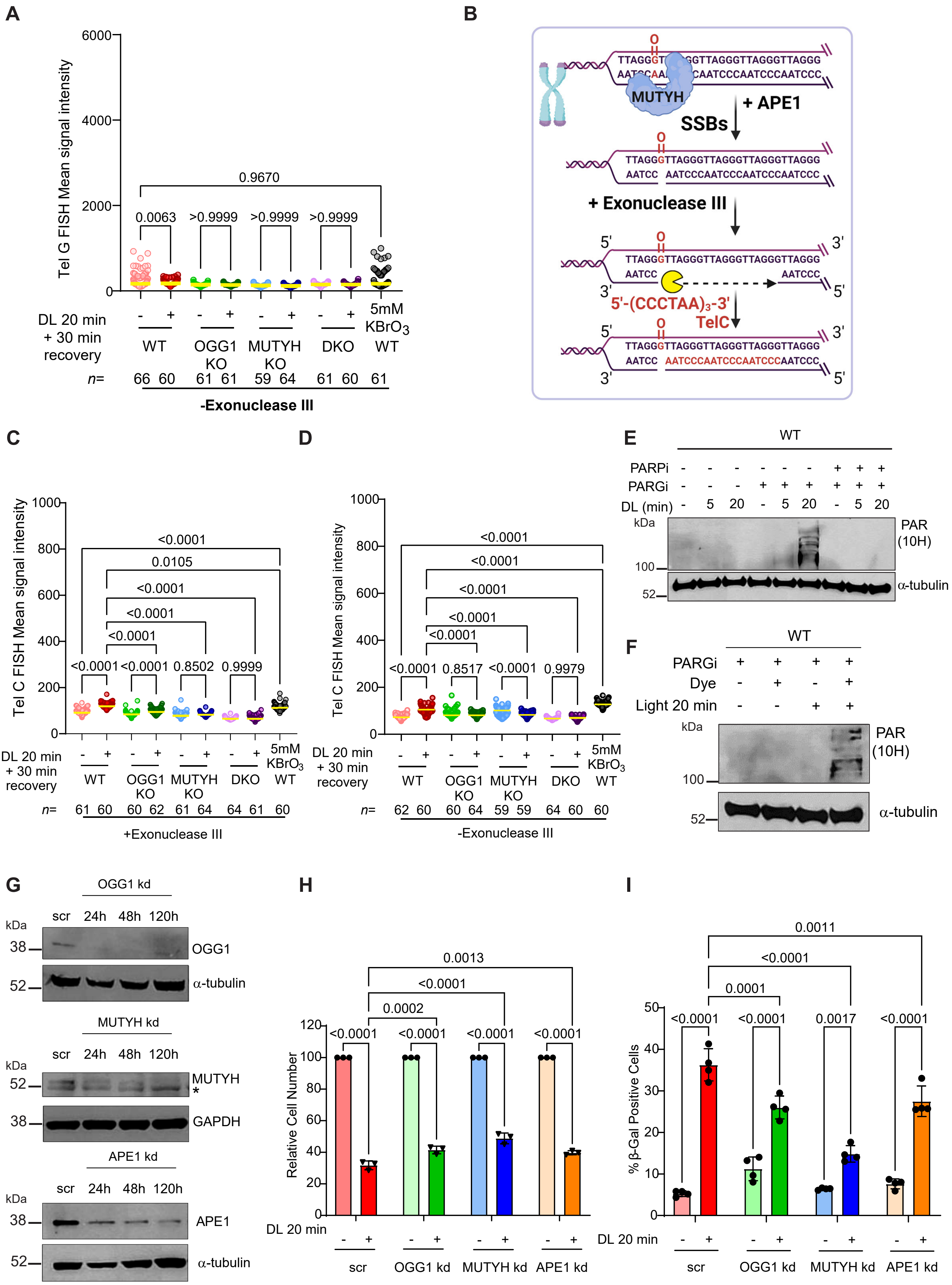
A
Tel G FISH Mean signal intensity
6000
4000
2000
0
0.9670
0.0063
>0.9999
>0.9999
>0.9999
DL 20 min
+ 30 min
recovery
- + - + - + - +
WT
OGG1 KO
MUTYH KO
DKO
5mM KBrO3 WT
n= 66 60 61 61 59 64 61 60 61
-Exonuclease III
B
TTAGGGTTAGGGTTAGGGTTAGGGTTAGGG
MUTYH
+ APE1
SSBs
TTAGGGTTAGGGTTAGGGTTAGGGTTAGGG
AATCC AATCCCAATCCCAATCCCAATCCC
+ Exonuclease III
5'
3'
AATCC
AATCCC
5'-(CCCTAA)3-3'
TelC
C
Tel C FISH Mean signal intensity
1000
800
600
400
200
0
<0.0001
0.0105
<0.0001
<0.0001
<0.0001
<0.0001 <0.0001 0.8502 0.9999
DL 20 min
+ 30 min
recovery
WT
OGG1 KO
MUTYH KO
DKO
5mM KBrO3 WT
n= 61 60 60 62 61 64 64 61 60
+Exonuclease III
D
Tel C FISH Mean signal intensity
<0.0001
<0.0001
<0.0001
<0.0001
<0.0001
<0.0001 0.8517 <0.0001 0.9979
DL 20 min
+ 30 min
recovery
WT
OGG1 KO
MUTYH KO
DKO
5mM KBrO3 WT
n= 62 60 60 64 59 59 64 60 60
-Exonuclease III
E
WT
PARPi - - - - - - + + +
PARGi - - - + + + + + +
DL (min) - 5 20 - 5 20 - 5 20
kDa
100
52
PAR (10H)
α-tubulin
F
WT
PARGi + + + +
Dye - + - +
Light 20 min - - + +
kDa
100
52
PAR (10H)
α-tubulin
G
OGG1 kd
scr 24h 48h 120h
kDa
38
52
OGG1
α-tubulin
MUTYH kd
scr 24h 48h 120h
kDa
52
38
MUTYH
*
GAPDH
APE1 kd
scr 24h 48h 120h
kDa
38
52
APE1
α-tubulin
H
Relative Cell Number
120
100
80
60
40
20
0
0.0013
<0.0001
0.0002
<0.0001 <0.0001 <0.0001 <0.0001
DL 20 min
- + - + - + - +
scr
OGG1 kd
MUTYH kd
APE1 kd
I
% β-Gal Positive Cells
50
40
30
20
10
0
0.0011
<0.0001
0.0001
<0.0001 <0.0001 0.0017 <0.0001
DL 20 min
- + - + - + - +
scr
OGG1 kd
MUTYH kd
APE1 kd

Supplementary Figure 4. BER intermediates promote telomeric 8oxoG-induced senescence. Related to Fig. 4.

A Quantification of Tel G exo-FISH signal intensity from controls without exonuclease III (buffer only). Each data point represents the mean fluorescence intensity value for each cell. Data represent the median of n = total number of analyzed nuclei from three independent experiments. P-values obtained using ordinary one-way ANOVA.

B Schematic of SSB repair intermediate detection from MUTYH processing of adenine misinserted opposite 8oxoG at telomeres using exo-FISH. Created in BioRender. De Rosa, M. (2024) https://BioRender.com/j57y455

C-D Quantification of TelC exo-FISH signal intensity from cells treated with exonuclease III (**C**) or buffer only (**D**). Each data point represents the mean fluorescence intensity for each cell. Data represent the median of n = total number of analyzed nuclei from three independent experiments. P-values obtained using ordinary one-way ANOVA.

E Immunoblot of poly-ADP ribose (PAR) in WT BJ FAP-TRF1 treated with 10 µM PARG inhibitor PDD 00017272 (PARGi), 10 µM PARP1 inhibitor AZD2281 (PARPi) and DL as indicated, for the indicated times. α-tubulin used as a loading control.

F Immunoblot of PAR in WT BJ FAP-TRF1 cells treated with 10 µM PARGi and dye only, 20 min of light only, or 20 min DL. α-tubulin used as a loading control.

G Immunoblot of OGG1, MUTYH or APE1 as indicated, at the indicated times following treatment of WT BJ FAP-TRF1 cells with targeting siRNA against OGG1, MUTYH and APE1, respectively. * symbol indicates a non-specific band below MUTYH. α-tubulin or GAPDH used as loading controls.

H-I Cells were treated with targeting siRNA to knock down (kd) OGG1, MUTYH or APE1, and then 24 h later treated with 20 min DL. (**H**) Counts of WT cells obtained 4 days after recovery from 20 min DL, relative to untreated cells. Data are mean ± SD from three independent experiments; P-values obtained using two-way ANOVA. (**I**) Percent β-galactosidase positive cells 4 days after recovery from DL 20 min. Data are mean ± SD from four independent experiments. P-values obtained using two-way ANOVA.

Source data provided as a Source Data file.

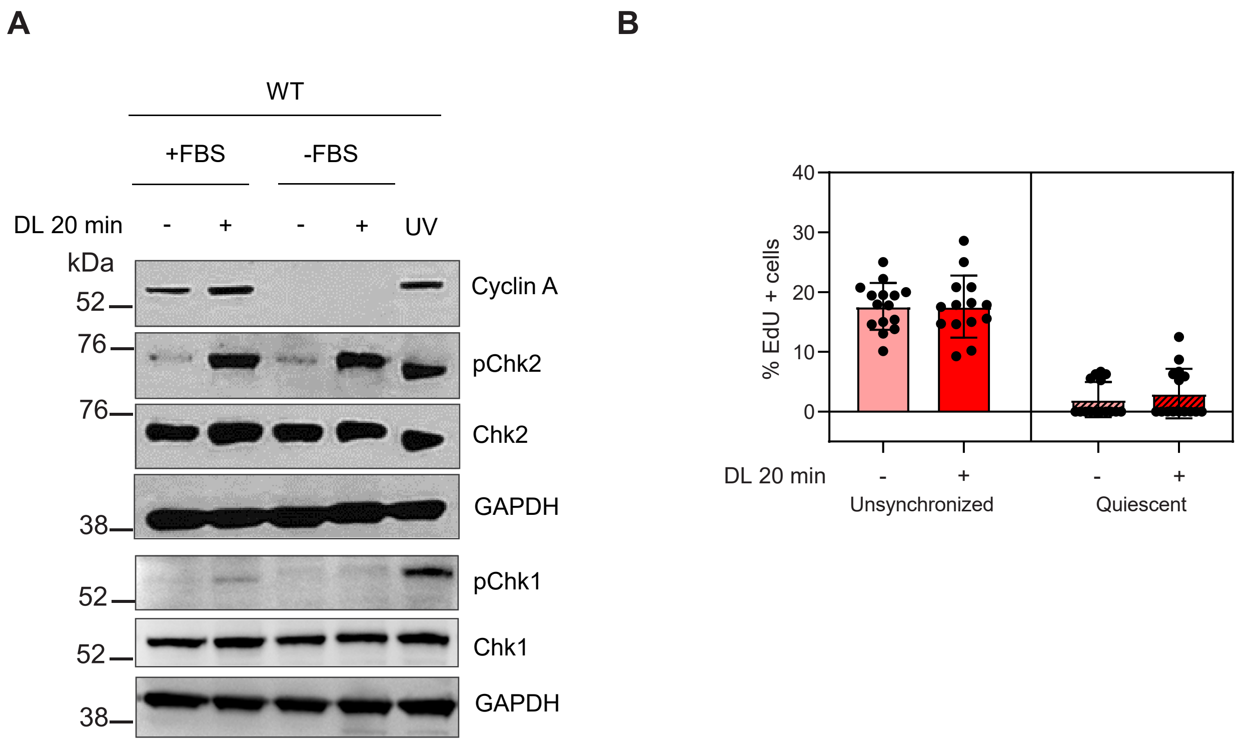

A
B
WT
+FBS
-FBS
DL 20 min
-
+
-
+
UV
kDa
52
76
76
38
52
52
38
Cyclin A
pChk2
Chk2
GAPDH
pChk1
Chk1
GAPDH
% EdU + cells
40
30
20
10
0
DL 20 min
-
+
-
+
Unsynchronized
Quiescent

Supplementary Figure 5. Both BER and replication stress activate PARylation after 8oxoG damage.

Related to Fig. 5.

A Immunoblot of Cyclin A, phosphorylated Chk2 (pChk2), Chk2, phosphorylated Chk1 (pChk1), and Chk1 in WT cells untreated or treated with 20 min DL or 20 J/m^2 UVC light as a positive control, and recovered 3 h. GAPDH used as a loading control.

B Quantification of percent EdU-positive cells in WT cells 3 hours after 20 min DL in 10% FBS (unsynchronized) or 0.1% FBS (quiescent). Error bars represent the mean ± SD from the number of independent random image fields scored.

Source data are provided as a Source Data file.

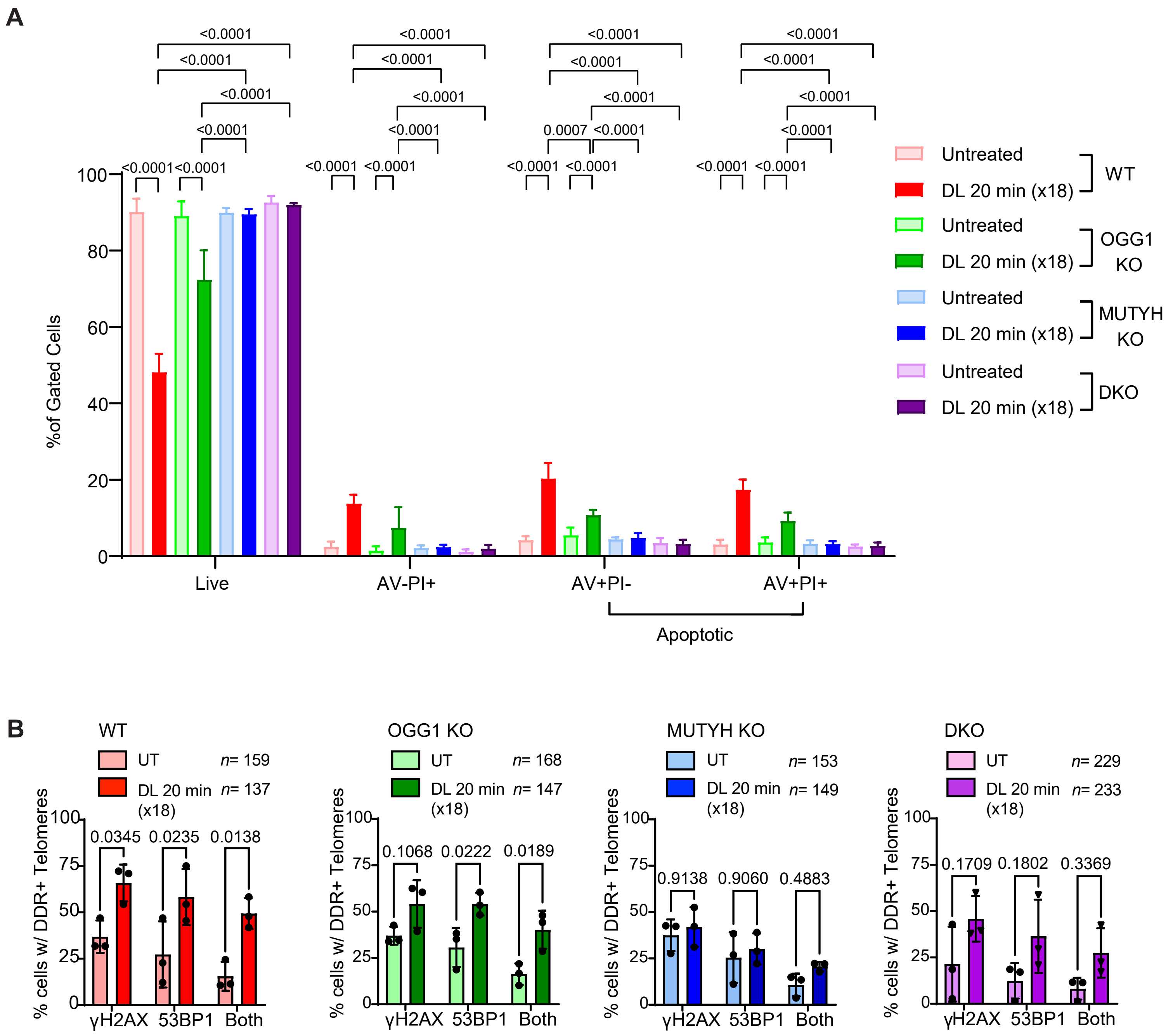
A
%of Gated Cells
Live
AV-PI+
AV+PI-
AV+PI+
Apoptotic
<0.0001
0.0007
Untreated
DL 20 min (x18)
WT
OGG1 KO
MUTYH KO
DKO
B
WT
OGG1 KO
MUTYH KO
DKO
UT
DL 20 min (x18)
n= 159
n= 137
n= 168
n= 147
n= 153
n= 149
n= 229
n= 233
% cells w/ DDR+ Telomeres
γH2AX
53BP1
Both
0.0345
0.0235
0.0138
0.1068
0.0222
0.0189
0.9138
0.9060
0.4883
0.1709
0.1802
0.3369

Supplementary Figure 6. OGG1 loss sensitizes cells to chronic telomere damage while MUTYH loss promotes resistance. Related to Fig. 6.

A Percent of cells positive for annexin V (AV), propidium iodide (PI), or both, 48h after 18x treatments of 20 min DL exposures. Data are mean ± SD from two independent experiments; *P*-values were obtained using two-way ANOVA.

B Quantification of the percentage of cells exhibiting telomere foci co-localized with γH2AX, 53BP1 or both for the indicated BJ FAP-TRF1 cell lines 24 h after 18x treatments of 20 min DL exposures. Data are the mean ± SD from three independent experiments of *n* total nuclei analyzed per condition. *P*-values were obtained using two-way ANOVA.

Source data are provided as a Source Data file.

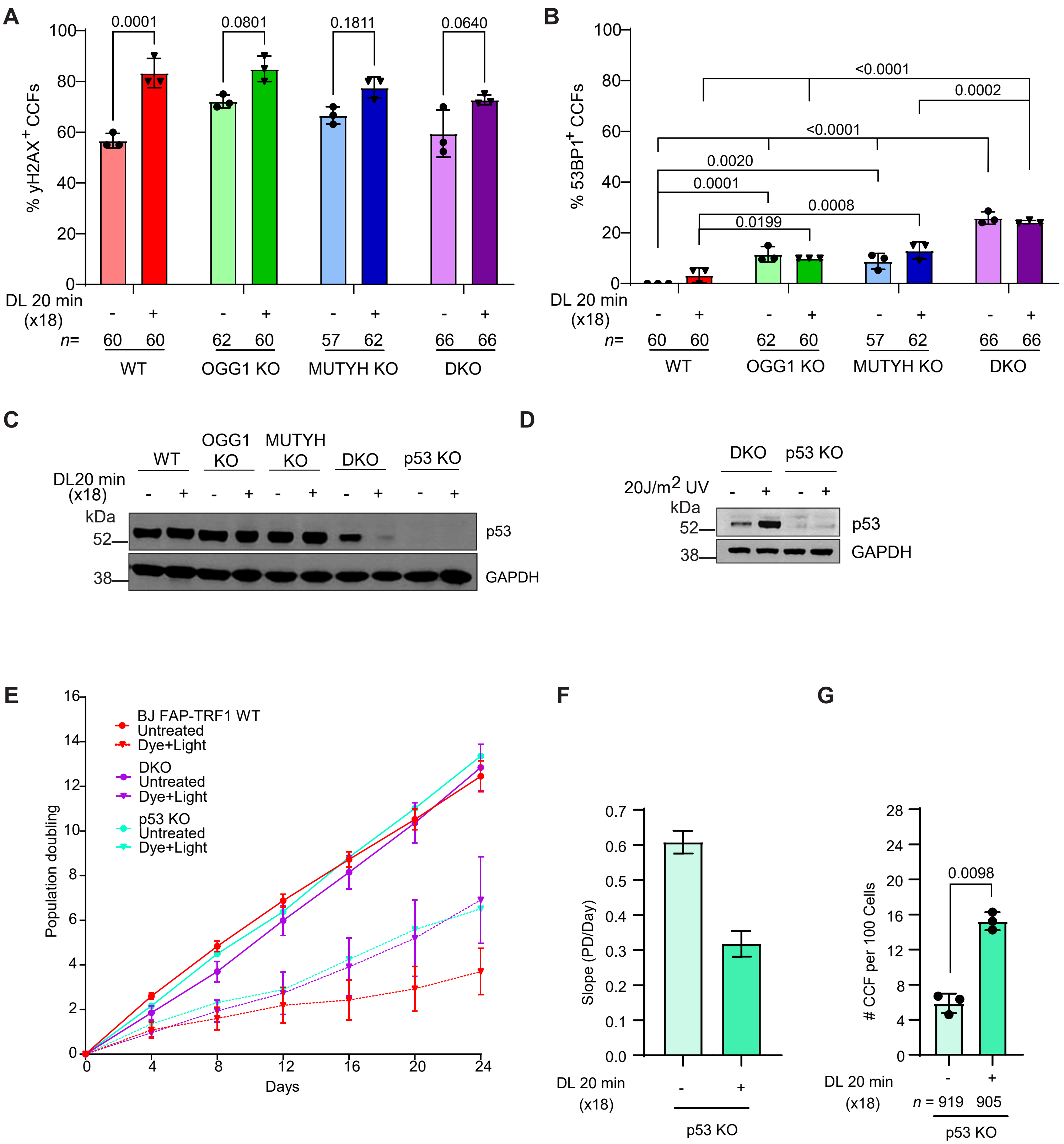

A
% γH2AX+ CCFs
0.0001
0.0801
0.1811
0.0640
DL 20 min (x18)
- + - + - + - +
n= 60 60 62 60 57 62 66 66
WT
OGG1 KO
MUTYH KO
DKO
B
% 53BP1+ CCFs
<0.0001
0.0002
<0.0001
0.0020
0.0001
0.0008
0.0199
DL 20 min (x18)
- + - + - + - +
n= 60 60 62 60 57 62 66 66
WT
OGG1 KO
MUTYH KO
DKO
C
WT
OGG1 KO
MUTYH KO
DKO
p53 KO
DL20 min (x18)
- + - + - + - + - +
kDa
52
38
p53
GAPDH
D
DKO
p53 KO
20J/m2 UV
- + - +
kDa
52
38
p53
GAPDH
E
BJ FAP-TRF1 WT
Untreated
Dye+Light
DKO
Untreated
Dye+Light
p53 KO
Untreated
Dye+Light
Population doubling
Days
F
Slope (PD/Day)
DL 20 min (x18)
- +
p53 KO
G
CCF per 100 Cells
0.0098
DL 20 min (x18)
- +
n = 919 905
p53 KO

Supplementary Figure 7. Chronic telomere damage drives chromosomal instability in OGG1 and MUTYH doubly deficient cells.

A-B Quantification of the percent of cytoplasmic DNA species CCF positive for DDR markers γH2AX (**A**) or 53BP1 (**B**) for the indicated cell lines. Data are mean ± SD from three independent experiments; *n* = total number of CCFs analyzed. *P*-values were obtained using two-way ANOVA.

C Immunoblot of p53 in cells untreated or treated with 20 min DL for 18 times and recovered 3h after the last treatment. GAPDH used as a loading control.

D Immunoblot of p53 in cells untreated or treated with 20J/m^2 UVC and recovered 3h after culturing for one month to mimic a chronic experiment. GAPDH used as a loading control.

E PD over 24 days of untreated cells (solid line) and cells treated with DL for 20 min each day except every 4th day of harvest (dotted line). Data for WT and DKO are mean ± SD from four independent experiments, and for p53 KO from three experiments.

F Slope of simple linear regression (PD/Day) of p53 KO cells treated 18 times with 20 min DL derived from three independent experiments shown in Panel **E**. Error bars represent the 95% confidence intervals of the slope estimate, with center indicating the slope of the regression line.

G Quantification of CCF 24 h after recovery from last exposure to 20 min DL (x18). Data are mean ± SD from three independent experiments, of *n*= total number of nuclei scored per condition; *P*-value was obtained using two-tailed paired t-test.

Source data are provided as a Source Data file.
