# Supplementary material for: OGG1 and MUTYH repair activities promote telomeric 8-oxoguanine induced senescence in human fibroblasts
Source: Nat Commun. 2025 Jan 21;16:893. doi: 10.1038/s41467-024-55638-4 (PMC11751180; doi:10.1038/s41467-024-55638-4)
Supplement: Supplementary file 2 — Reporting Summary [file 41467_2024_55638_MOESM2_ESM.pdf]

nature portfolio

Corresponding author(s): Patricia Opresko

Last updated by author(s): Nov 26, 2024

Reporting Summary

Nature Portfolio wishes to improve the reproducibility of the work that we publish. This form provides structure for consistency and transparency in reporting. For further information on Nature Portfolio policies, see our Editorial Policies and the Editorial Policy Checklist.

Statistics

For all statistical analyses, confirm that the following items are present in the figure legend, table legend, main text, or Methods section.

n/a	Confirmed	
	x	The exact sample size (*n*) for each experimental group/condition, given as a discrete number and unit of measurement
	x	A statement on whether measurements were taken from distinct samples or whether the same sample was measured repeatedly
	x	The statistical test(s) used AND whether they are one- or two-sided<br>*Only common tests should be described solely by name; describe more complex techniques in the Methods section.*
x		A description of all covariates tested
x		A description of any assumptions or corrections, such as tests of normality and adjustment for multiple comparisons
	x	A full description of the statistical parameters including central tendency (e.g. means) or other basic estimates (e.g. regression coefficient) AND variation (e.g. standard deviation) or associated estimates of uncertainty (e.g. confidence intervals)
	x	For null hypothesis testing, the test statistic (e.g. *F*, *t*, *r*) with confidence intervals, effect sizes, degrees of freedom and *P* value noted<br>*Give P values as exact values whenever suitable.*
x		For Bayesian analysis, information on the choice of priors and Markov chain Monte Carlo settings
x		For hierarchical and complex designs, identification of the appropriate level for tests and full reporting of outcomes
x		Estimates of effect sizes (e.g. Cohen's *d*, Pearson's *r*), indicating how they were calculated

Our web collection on statistics for biologists contains articles on many of the points above.

Software and code

Policy information about availability of computer code

Data collection	Nikon NIS Elements AR 5.02.01 for microscopy images.
Data analysis	GraphPad Prism Software, Version 10.

For manuscripts utilizing custom algorithms or software that are central to the research but not yet described in published literature, software must be made available to editors and reviewers. We strongly encourage code deposition in a community repository (e.g. GitHub). See the Nature Portfolio guidelines for submitting code & software for further information.

Data

Policy information about availability of data

All manuscripts must include a data availability statement. This statement should provide the following information, where applicable:

- Accession codes, unique identifiers, or web links for publicly available datasets
- A description of any restrictions on data availability
- For clinical datasets or third party data, please ensure that the statement adheres to our policy

All data generated or analyzed during this study are included in this published article and its supplementary information files. Source data are provided with this paper.

Research involving human participants, their data, or biological material

Policy information about studies with human participants or human data. See also policy information about sex, gender (identity/presentation), and sexual orientation and race, ethnicity and racism.

Reporting on sex and gender	N/A
Reporting on race, ethnicity, or other socially relevant groupings	N/A
Population characteristics	N/A
Recruitment	N/A
Ethics oversight	N/A

Note that full information on the approval of the study protocol must also be provided in the manuscript.

Field-specific reporting

Please select the one below that is the best fit for your research. If you are not sure, read the appropriate sections before making your selection.

☒ Life sciences ☐ Behavioural & social sciences ☐ Ecological, evolutionary & environmental sciences

For a reference copy of the document with all sections, see nature.com/documents/nr-reporting-summary-flat.pdf

Life sciences study design

All studies must disclose on these points even when the disclosure is negative.

Sample size	As is standard in the field, in immunofluorescence (IF) experiments at least 40-100 nuclei were counted per condition.<br>As is standard in the field, at least 20 metaphases were analyzed per conditions in all metaphase spreads Fluorescence In Situ Hybridization (FISH) analyses.<br>For cytoplasmic chromatin fragments (CCFs) counting, at least 300-400 nuclei were counted per condition.<br>For CCFs content analysis by IF-FISH, at least 20 nuclei were counted per condition.<br>For chromatin bridges counting, at least 1000 nuclei were counted per condition.<br>For Exo-FISH experiments, at least 20 nuclei were analyzed per condition.<br>Each experiment was performed at least 3 times independently. These information are specified in the figure legends.
Data exclusions	No data were excluded from any analysis.
Replication	Each experiment was replicated at least 3 times with successful reproducibility as is specified in the figure legends.
Randomization	Samples were allocated into the group depending on cell treatments as described in the manuscript. Randomization was not employed during these experiments because of the relatively small group numbers.
Blinding	Where possible (e.g. IF, metaphase analyses, CCFs and bridges counting) samples were scored blindly. Before slide mounting, the person taking care of that specific experiment would attribute numbers to each sample by randomly applying an adhesive tag covering the original label, and would then reconnect the results to the experimental condition after the analysis was performed.<br>For other experiments (e.g. western blot, or beta galactosidase staining acquisition), blinding was not possible because samples were respectively loaded onto gels, or cells were seeded in six-wells, in logical ways that require the experimentalist to know the treatment conditions.

Reporting for specific materials, systems and methods

We require information from authors about some types of materials, experimental systems and methods used in many studies. Here, indicate whether each material, system or method listed is relevant to your study. If you are not sure if a list item applies to your research, read the appropriate section before selecting a response.

Materials & experimental systems

n/a	Involved in the study
☐	☒ Antibodies
☐	☒ Eukaryotic cell lines
☒	☐ Palaeontology and archaeology
☒	☐ Animals and other organisms
☒	☐ Clinical data
☒	☐ Dual use research of concern
☒	☐ Plants

Methods

n/a	Involved in the study
☒	☐ ChIP-seq
☐	☒ Flow cytometry
☒	☐ MRI-based neuroimaging

Antibodies

Antibodies used

Anti-MUTYH mouse monoclonal Abnova Cat#H00004595-M01; RRID:AB_540648
Anti-OGG1 rabbit monoclonal Abcam Cat#ab124741; RRID:AB_10973360
Anti-alpha-tubulin mouse monoclonal Millipore Cat#05-829; RRID:AB_310035
Anti-TRF1 (TRF-78) mouse monoclonal Santa Cruz Cat#sc-56807 trf1-antibody-trf-78; RRID:AB_793407
Anti-beta-actin mouse monoclonal Cell Signaling Cat#3700; RRID:AB_2242334
Anti-gammaH2AX (Ser139) mouse monoclonal Santa Cruz Cat#sc-517348 p-histone-h2a-x-antibody ser-139; RRID:AB_2783871
Anti-53BP1 rabbit polyclonal Novus Cat#NB100-304; RRID:AB_10003037
Anti-cGAS rabbit monoclonal Cell Signaling Cat#15102; RRID:AB_2732795
Anti-Phospho-Chk1 (ser345) rabbit monoclonal Cell Signaling Cat#2348 phospho-chk1-ser345-133d3; RRID:AB_331312
Anti-Chk1 (2G1D5) mouse monoclonal Cell Signaling Cat#2360 chk1-2g1d5-mouse-mab; RRID:AB_2080320
Anti-GAPDH mouse monoclonal Santa Cruz Cat#sc-47724; RRID:AB_627678
Ant-Phospho-ATM (ser1981) rabbit monoclonal Abcam Cat#ab81292; RRID:AB_1640207
Anti-ATM mouse monoclonal Sigma Cat#A1106; RRID:AB_796190
Anti-Phospho-Chk2 (Thr68) rabbit monoclonal Cell Signaling Cat#2197 anti phospho-chk2; RRID: AB_2080501
Anti-Chk2 mouse monoclonal Cell Signaling Cat#3440 chk2-1c12-mouse-mab; RRID:AB_2229490
Anti-p21 rabbit monoclonal Cell Signaling Cat#2947; RRID:AB_823586
Anti-p53 mouse monoclonal Santa Cruz Cat#sc-126; RRID:AB_628082
Anti-Poly(ADP-ribose) (10H) mouse monoclonal Enzo Life Sciences Cat# ALX-804-220-R100; RRID:AB_2052275
Anti-APE1 rabbit monoclonal Cell Signaling Cat#10519S; N/A
Anti-Cyclin A mouse monoclonal Santa Cruz Cat#sc-271682; RRID:AB_10709300
Anti-Phospho-STING (Ser366) rabbit monoclonal Cat#19781; RRID:AB_2737062
Anti-STING rabbit monoclonal Cat#13647; RRID:AB_2732796

Validation

All antibodies are commercially available and validated by the manufacturers. The species are mentioned above and the application can be accessed on the manufacturers' websites. The validation of these antibodies has been confirmed on human lysates using western blot and immuno-fluorescence according to the product instructions. Below are the links:
Abnova: “Guarantee for all products manufactured by Abnova” https://www.abnova.com/en-global/staticpage/100percent
Abcam: "Our Abpromise guarantee covers the use of ab12474/ab81292 in the following tested applications" https://www.abcam.com/primary-antibodies/how-we-validate-our-antibodies
Millipore/Sigma: "MilliporeSigma's highly validated antibodies are guaranteed for quality performance. In addition to application-specific validation, all of our antibodies are backed with a best-in-industry technical service team dedicated to our antibodies customers. These efforts and collaborations have led to new validation techniques and novel antibody-based technologies, such as improved bead-based multiplex assays and imaging flow cytometry"
https://www.emdmillipore.com/US/en/life-science-research/antibodies-assays/antibodies-overview/Antibody-Development-and-Validation/cFOb.qB.8McAAAFOb64qQvSS,nav
Santa Cruz: “Santa Cruz Biotechnology is committed to providing the highest level of quality and service”. https://www.scbt.com/customer-care/terms
Cell Signaling: “To ensure our antibodies will work in your experiment, we adhere to the Hallmarks of Antibody Validation™, six complementary strategies that can be used to determine the functionality, specificity, and sensitivity of an antibody in any given assay. CST adapted the work by Uhlen, et. al., (“A Proposal for Validation of Antibodies.” Nature Methods (2016)) to build the Hallmarks of Antibody Validation, based on our decades of experience as an antibody manufacturer and our dedication to reproducible science”. https://www.cellsignal.com/about-us/cst-antibody-validation-principles
Novus Biologicals: "Novus Biologicals Is a Trusted Leader in Quality Life Science Reagents. We Are Committed to Providing Researchers With the Highest Quality Antibodies" "To that end, we actively seek high quality, highly validated products and provide support to ensure that our customers have the tools to properly validate their own assays. We are also collaborating with several global initiatives that help life science researchers choose antibodies with proven results. Of the five pillars of validation established by these initiatives, genetic knockout validation provides the most reliable control for assessing antibody specificity"
https://www.novusbio.com/reproducibility.html
Enzo Life Sciences: "monoclonal and polyclonal antibodies [are] backed by peer-reviewed citations, our Worry-free Antibody Trial Program and expert technical support"
https://www.enzolifesciences.com/browse/products/by-product-type/antibodies/
ThermoFisher: "To drive reagent quality, directly address antibody reproducibility, and help provide customers with confidence in our antibodies, Thermo Fisher Scientific has adopted three concepts to help improve experimental reproducibility and reporting based on the recommendations of the International Working Group for Antibody Validation (IWGAV):https://www.thermofisher.com/us/en/

home/life-science/antibodies/invitrogen-antibody-validation.html"

Eukaryotic cell lines

Policy information about cell lines and Sex and Gender in Research

Cell line source(s)	All our cell lines are derived from the BJhTERT expressing telomere FAP developed in Dr. Opresko lab as described previously: Barnes et al., NSMB 2022.
Authentication	Cell lines were authenticated previously (Barnes et al., NSMB 2022) . For derivatives of these cells generated in this study, the protein levels of the knocked out genes were established by western blotting with appropriate antibodies.
Mycoplasma contamination	All our cell Lines are tested for mycoplasma contamination on a monthly basis using the ATCC Universal Mycoplasma Detection Kit to confirm that they test negative for mycoplasma infection. All cells lines used in this study tested negative for mycoplasma infection.
Commonly misidentified lines (See ICLAC register)	No commonly misidentified lines were used in this study.

Plants

Seed stocks	*Report on the source of all seed stocks or other plant material used. If applicable, state the seed stock centre and catalogue number. If plant specimens were collected from the field, describe the collection location, date and sampling procedures.*
Novel plant genotypes	*Describe the methods by which all novel plant genotypes were produced. This includes those generated by transgenic approaches, gene editing, chemical/radiation-based mutagenesis and hybridization. For transgenic lines, describe the transformation method, the number of independent lines analyzed and the generation upon which experiments were performed. For gene-edited lines, describe the editor used, the endogenous sequence targeted for editing, the targeting guide RNA sequence (if applicable) and how the editor was applied.*
Authentication	*Describe any authentication procedures for each seed stock used or novel genotype generated. Describe any experiments used to assess the effect of a mutation and, where applicable, how potential secondary effects (e.g. second site T-DNA insertions, mosiacism, off-target gene editing) were examined.*

Flow Cytometry

Plots

Confirm that:

☒ The axis labels state the marker and fluorochrome used (e.g. CD4-FITC).

☒ The axis scales are clearly visible. Include numbers along axes only for bottom left plot of group (a 'group' is an analysis of identical markers).

☒ All plots are contour plots with outliers or pseudocolor plots.

☒ A numerical value for number of cells or percentage (with statistics) is provided.

Methodology

Sample preparation	Cells were treated as described and assayed for Annexin V / Propidium Iodide staining with the Dead Cell Apoptosis Kit from ThermoFisher.
Instrument	CytoFLEX S Flow Cytometer (Beckman)
Software	FlowJo v10.8.1
Cell population abundance	At least 20000 events were collected per condition after
Gating strategy	For cell apoptosis detection, AnnexinV–PI–, AnnexinV–PI+, AnnexinV+PI–, AnnexinV+PI+ staining represents viable cells, necrotic cells, early apoptotic cells, and late apoptosis cells being stained, respectively. PBS-treated cells were stained with/without Annexin-V-FITC or PI to determine gate.

☒ Tick this box to confirm that a figure exemplifying the gating strategy is provided in the Supplementary Information.
